# Noninfectious Necrotizing Aortitis With Predominantly Neutrophilic Infiltrate

**DOI:** 10.1155/crip/5727208

**Published:** 2026-01-08

**Authors:** Nathan A. Williams, Mark Colantonio, Anastasia Miller, Allen P. Burke

**Affiliations:** ^1^ Department of Pathology, University of Maryland School of Medicine, Baltimore, Maryland, USA, umaryland.edu; ^2^ Department of Internal Medicine, West Virginia University School of Medicine, Baltimore, Maryland, USA, wvu.edu

## Abstract

Aortitis can be either infectious or noninfectious, and cases of noninfectious aortitis are not well understood. Our study presents a case of noninfectious ascending aortitis in a relatively young, previously healthy man, characterized by a predominantly neutrophilic infiltrate on histopathological examination without an inciting microorganism. This case report highlights that a neutrophilic infiltrate does not necessarily indicate an infectious etiology in ascending aortic aneurysms.

## 1. Introduction

Aortitis can present in many different ways and encompasses a wide spectrum of pathological findings, including infectious or noninfectious etiologies [1]. Commonly, cases of aortitis are due to large vessel vasculitides, including giant cell arteritis (GCA) and Takayasu arteritis. Historically, infectious aortitis was most commonly secondary to *Treponema pallidum* and primarily affecting the ascending aorta. Infectious aortitis is most frequently a complication of bacteremia and often associated with endocarditis, leading to fever and leukocytosis [2]. Infectious aortitis is mostly seen in the descending thoracic aorta. Etiologies include *Staphylococcus aureus*, *Streptococcus*, and other Gram‐negative bacilli [3]. Histological evaluation can be essential for differntiating infectious versus noninfectious cases of aortitis. Ramiro et al. described a case of infectious aortitis in an elderly female with evidence of neutrophilic infiltrates on vascular wall histology, whereas Miller et al. conducted a retrospective review of 45 cases of confirmed noninfectious aortitis and described chronic inflammation characterized by the presence of multinucleated giant cells and mononuclear cells, including lymphocytes and macrophages [4, 5].

The etiology of noninfectious aortitis remains poorly understood and is often presumed to be autoimmune in etiology [6]. Noninfectious aortitis can be classified into several major disease classifications, while giant cell/granulomatous aortitis (including Takayasu aortitis) is the most common [6]. Other etiologies include lymphoplasmacytic aortitis (including IgG4‐related sclerosing and rheumatologic disease), granulomatosis with polyangiitis (GPA), sarcoidosis, Cogan syndrome, and Behçet disease [6]. Most cases of noninfectious aortitis are incidental and represent 6% of ascending aortic aneurysm repairs [7].

Literature has outlined four main histological patterns of aortitis, including granulomatous or giant cell pattern, lymphoplasmacytic pattern, mixed inflammatory pattern, and suppurative pattern [8]. However, noninfectious aortitis with a predominantly neutrophilic inflammatory response has not been reported. We report a case of noninfectious ascending aortitis with suppurative features, resulting in the absence of an inciting microorganism. Pathologists and treating physicians should be aware that a neutrophilic infiltrate does not necessarily indicate an infectious etiology when associated with an ascending aortic aneurysm.

## 2. Case

A 25‐year‐old male presented to the emergency department (ED) with worsening chest pain radiating to his left arm, mild tachycardia, and new T wave inversions on EKG. In the ED, the patient was febrile to 102.8°F, with a heart rate in the 110–120 s, respiratory rate in the 30 s, blood pressure in the 120 s/70 s, and oxygen saturation of 99% on room air. Laboratory evaluation revealed a white blood cell (WBC) count of 23.6, hemoglobin of 10, platelets of 614, and an unremarkable basic metabolic panel (BMP). Liver enzymes were elevated with an aspartate aminotransferase (AST) of 105 and an alanine aminotransferase (ALT) of 91. Troponin was < 0.01. Coagulation studies showed a prothrombin time (PT) of 16, an international normalized ratio (INR) of 1.4, and a partial thromboplastin time (PTT) of 50. Imaging revealed a new left lower lobe infiltrate, a moderate pericardial effusion, and a 1.8 × 0.8 cm cavitary lesion in the left upper lobe. He was started on vancomycin and piperacillin–tazobactam and subsequently underwent bronchoscopy, during which bronchoalveolar lavage (BAL) was positive for Coxsackie virus. He was discharged 6 days after a negative infectious and rheumatologic workup, including negative blood cultures. Rapid plasmin reagin (RPR) was also negative. Additional serologies were negative for *Coxiella*, *Bartonella*, and *Brucella*; viral serologies were negative except for Coxsackie virus, which partially explained the pericarditis. BAL cultures obtained for evaluation of the cavitary lung lesion were negative for acid‐fast bacillus (AFB) and fungal organisms. Mycoplasma polymerase chain reaction (PCR) and Nocardia culture were also negative. For the rheumatologic workup, antinuclear antibody (ANA) and double‐stranded DNA were negative; C3 and C4 were within normal limits; c‐anti‐neutrophil cytoplasmic antibody (ANCA) and p‐ANCA were negative; erythrocyte sedimentation rate (ESR) was 50; and both rheumatoid factors and anti‐CCP were normal.

One week later, he re‐presented with chest pain, dyspnea, and fevers and was found to have a 5 cm ascending aortic aneurysm on computed tomography (CTA). After stabilization and initiation of antibiotics, he was assessed by cardiac surgery. He underwent emergent ascending aorta and hemiarch replacement 1 day after, with a subsequent re‐exploration for bleeding and eventual chest closure. Infectious disease was consulted postoperatively for persistent fevers and a cavitary lung lesion, with concern for a mycotic aneurysm. He was treated with vancomycin and ceftriaxone, and 16s PCR from aortic tissue was sent. Histopathology revealed transmural purulent inflammation with a predominance of neutrophils and necrosis of the media, diagnostic of necrotizing aortitis. Elastic stains demonstrated extensive transmural destruction of the media. Special stains were negative, including Grocott‐methenamine silver (GMS) and AFB. Gram staining and blood cultures demonstrated light growth of *Staphylococcus hominis*, a common contaminant of typical skin flora. PCR results were negative for fungi (using 28S rDNA), bacteria (using 16s rDNA), and nontuberculous mycobacteria, including *Mycobacterium avium* complex (detected by 16s rDNA, hsp65, and rpoB). Additionally, tests for *Mycobacterium tuberculosis* complex DNA (using hsp65 amplified probe) were negative. Genetic screening for aortic diseases, including Marfan’s syndrome, Loeys–Dietz syndrome, and vascular Ehlers–Danlos syndrome, was also negative. After clinical improvement, negative blood cultures, and negative AFB smears, he was discharged 1 month from his admission date with a PICC line for continued IV antibiotics and outpatient infectious disease follow‐up (Supporting Information File S1).

## 3. Discussion

Aortitis with predominantly neutrophilic infiltrates is typically secondary to bacterial infection associated with an aortic pseudoaneurysm. Large numbers of neutrophils are rare in ascending aortitis and are usually associated with an autoimmune disease or syphilis. This case differs significantly from infectious pseudoaneurysms. It is worth mentioning that while *C. septicum* can occur, it is almost always associated with pseudoaneurysm formation and periaortic gas, which were not seen. Aortic dissections are an uncommon complication of noninfectious aortitis and typically occur in the lymphoplasmacytic type, which does not result in scarring, thereby preventing medial dissection.

In this case, PCR and special stains were negative for bacteria, fungi, and mycobacteria, consistent with findings on histopathological examination. It should be noted that the only organism that demonstrated growth in blood cultures was *Staphylococcus hominis*. This Gram‐positive, coagulase‐negative bacterium is part of the normal skin flora and is unlikely to cause the pathological features observed in this case. We recognize that, occasionally, PCR testing from the formalin‐fixed paraffin‐embedded method can yield low nucleic acid yields due to formalin fixation [9]. However, we have no evidence that the extracted tissue had a low nucleic acid yield. Additionally, we recognize that the patient was treated with antibiotics before readmission, which may have limited the detection of microorganisms. However, the diagnostics performed with the AFB, Gram, GMS, special stains, PCR, and the gold standard of blood cultures maximize the likelihood of detecting a microorganism at our tertiary academic center. Additionally, it has been reported that 16S rRNA has a 100% and 80% positive predictive and negative predictive value, respectively, in culture‐negative bacterial infections [10]. Also, given the clinical presentation, imaging, and histopathology, this is the best explanation. In summary, we propose a “neutrophilic” subtype of noninfectious aortitis to expand the histologic classification scheme of aortitis proposed by Stone et al. This case expands our current thinking about how morphological findings that show an infectious‐like pattern could result from a noninfectious cause.

## Consent

All the patients/next of kin allowed personal data processing, and informed consent was obtained from all individual participants included in the study.

## Conflicts of Interest

The authors declare no conflicts of interest.

## Funding

No funding was received for this manuscript.

## Supporting information


**Supporting Information** Additional supporting information can be found online in the Supporting Information section. File S1 The supporting file presents the CARE Case Report Guidelines Checklist to ensure completeness and transparency of the clinical information provided.

## Data Availability

The data that support the findings of this study are available on request from the corresponding author. The data are not publicly available due to privacy or ethical restrictions.
